# Does collagen cross linking have any effect on retinal circulation in patients with keratoconus? An optical coherence tomography angiography (OCTA) study

**DOI:** 10.1186/s12886-024-03470-1

**Published:** 2024-05-03

**Authors:** Shahram Bamdad, Alireza Attar, Milad Fallahzadeh, Fatemeh Ebrahimi, Sanam Faizabadi, Ali Azimi

**Affiliations:** Poostchi Ophthalmology Research Center, Department of Ophthalmology, School of Medicine, Shiraz University of Medical Sciences, Shiraz, Iran

**Keywords:** Collagen Cross linking, Keratoconus, Optical coherence tomography angiography

## Abstract

**Background:**

We aimed to employ Optical Coherence Tomography Angiography (OCTA) to comprehensively assess changes in the optic nerve head (ONH) and macular perfusion before and after the Corneal Collagen Cross-Linking (CCL) procedure in patients with keratoconus.

**Methods:**

A total of 22 keratoconus patient’s candidate for CCL procedures were included based on specific criteria, with meticulous exclusion criteria in place to minimize potential confounders. Participants underwent OCTA assessments of the ONH and macula using the Spectralis OCT (Heidelberg) before CCL, as well as at 1- and 3-months post-CCL. MATLAB software was utilized for image analysis.

**Results:**

The mean age of the participants was 20.09 ± 6.11, including 59% male, and the mean intraocular pressure (IOP) before the surgery was 13.59 ± 2.85 mmHg. Peripapillary Retinal nerve fiber layer (ppRNFL) thickness and overall retinal thickness remained stable post-CCL. However, significant alterations were observed in macular vessel density, emphasizing regional variations in vascular response. For macular large vessel density (LVD), both superficial and deep vascular complex (SVC and DVC) demonstrated significant differences between before surgery and the 3 months post-surgery follow-up (*p* < 0.001 and *p* = 0.002, respectively). Optic nerve head markers demonstrated relative stability, except for changes in avascular complex density, which was 49.2 ± 2.2% before the surgery and decrease to 47.6 ± 1.7% three months after the operation (*P*-value = 0.005).

**Conclusion:**

While CCL appears to maintain the integrity of certain ocular structures, alterations in macular perfusion post-CCL suggest potential effects on retinal blood supply. Long-term monitoring is crucial to understand the implications of these changes, particularly in the context of conditions such as diabetes.

## Background

Keratoconus (KCN) is a non-inflammatory condition characterized by bilateral and gradual ectasia, leading to corneal thinning and bulging [1]. Clinical observations of KCN often reveal progressive myopia and irregular astigmatism [2]. The reported figures for KCN’s prevalence and incidence vary, with recent data suggesting an annual incidence of 1 in 7,500 individuals (13.3 cases per 100,000) and an estimated prevalence of 1 in 375 people (265 cases per 100,000) [3]. Various treatment options are available for KCN, ranging from spectacles, toric soft contact lenses, and rigid gas-permeable lenses for less severe cases, to keratoplasty in situations of severe ectatic corneas, intolerance to contact lenses, or significant complications like hydrops and scarring [4–6]. Additionally, corneal collagen cross-linking (CCL) is employed when there is evidence of disease progression, involving the application of ultraviolet-A and riboflavin to the cornea to create chemical bonds between collagen fibers and impede disease advancement [7]. CCL is generally considered safe, with major complications being rare and primarily localized to the anterior segment of the eye [8, 9]. It’s worth noting that UVA radiation can potentially pose a greater risk to the retina compared to other wavelengths of light [10, 11]. Although in vitro research indicates that riboflavin shields inner ocular structures from radiation, there is a shortage of in vivo studies examining morphological changes in the retina following UVA-riboflavin CCL [12]. As a result, this study aimed to assess the impact of collagen crosslinking (CCL) on macula and optic nerve head circulation using OCT angiography in patients with keratoconus.

## Materials and methods

**Fig. 1:**
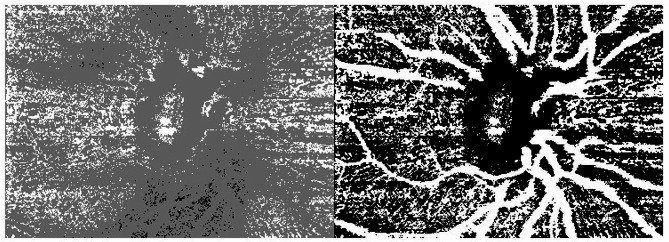
OCTA image of choroidal vascular density (CVD)

**Fig. 2:**
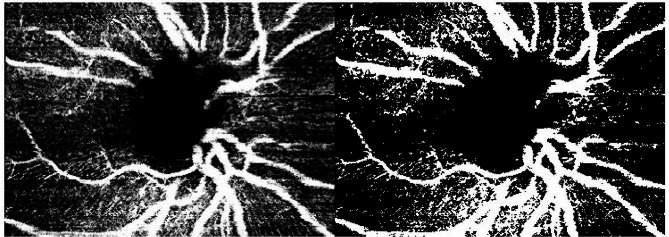
OCTA image of leaky vessel density (LVD)

**Fig. 3:**
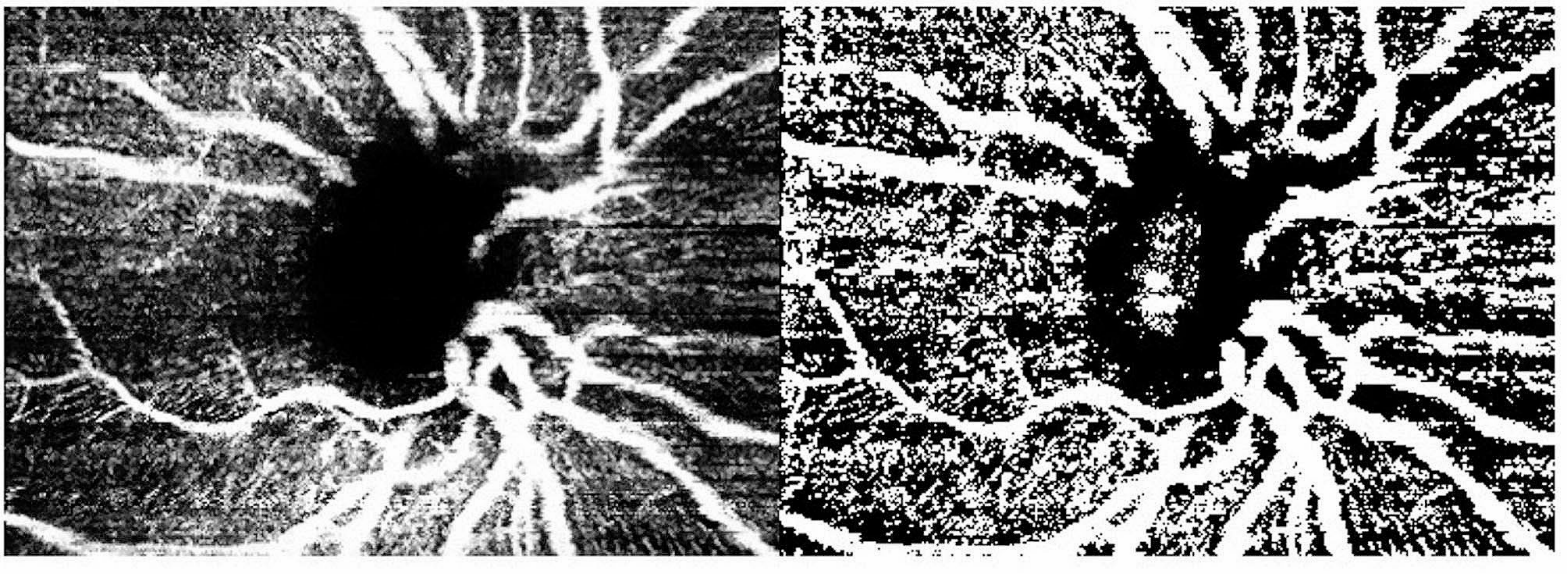
OCTA image of total vascular density (TVD)

This prospective cohort study aimed to comprehensively examine the effects of Corneal Collagen Cross-Linking (CCL) on Optic Nerve Head (ONH) and macular perfusion in 22 keratoconus patients, selected based on specific inclusion criteria. To maintain scientific rigor, stringent exclusion criteria have been established, excluding patients with central corneal thickness below 400 microns, a history of HSV keratitis, retinal or optic nerve disease, systemic conditions like diabetes and hypertension, or the use of retinally toxic drugs. Additionally, patients exhibiting significant corneal haze one-month post-Collagen Cross-Linking (CXL), which could potentially compromise the quality of image acquisition, were excluded from the study. However, individuals with subtle corneal haze that did not impair image quality were retained for investigation purposes. This meticulous design ensures that the study minimizes potential confounders, enabling robust and credible assessment of the role of CCL in improving ONH and macular perfusion in keratoconus patients. In this study, all participants from the target group underwent OCT angiography of the Optic Nerve Head (ONH) and macula, using the Spectralis OCT by Heidelberg. To minimize potential diurnal variations in retinal vascular circulation, all Optical Coherence Tomography Angiography (OCTA) measurements were conducted within the time frame of 9:00 AM to 11:00 AM. These assessments were conducted prior to the Corneal Collagen Cross-Linking (CCL) procedure and at 1- and 3-months post-CCL. Vascular density measurements in both the upper and deep layers of the macula and ONH were determined to evaluate changes resulting from CCL treatment. To obtain data and vessel densities, MATLAB software was employed to analyze OCTA images. Initially, an adaptive median filter was applied to reduce image noise. Subsequently, for Total Vascular Density (TVD), a local adaptive filter with a sensitivity of 0.7 was used. Global thresholding within the range of 0.45 to 0.49 was applied for analysis of Large Vessel Density (LVD). Binary outputs were obtained and used to calculate TVD and LVD by dividing the number of outputs by the total matrix arrays. Capillary Vascular Density (CVD) was derived by subtracting LVD from TVD (Figs. 1, 2 and 3).

### Cross-linking procedure

The standard Dresden protocol was followed for CCL [13]. In summary, after using topical anesthesia with Tetracaine hydrochloride 0.5%, the central 10 mm of the cornea was de-epithelialized, and a mixture of riboflavin phosphate 0.1% and dextran 20% was applied every 2 min for 30 min. UV-A irradiation at 370 nm and 3 mW/cm2 was applied every 5 min for 30 min.

### Optical coherence tomography

Macular retinal anatomy was assessed using spectral domain optical coherence tomography (SD-OCT) with a Heidelberg Engineering Spectralis HRA®OCT device. Patients received pupil dilation with eye drops and fixated on a target light. Foveal fixation was monitored with an infrared camera, and macular thickness in the central ring was measured using retinal mapping software.

### Optical coherence tomography angiography

**Fig. 4:**
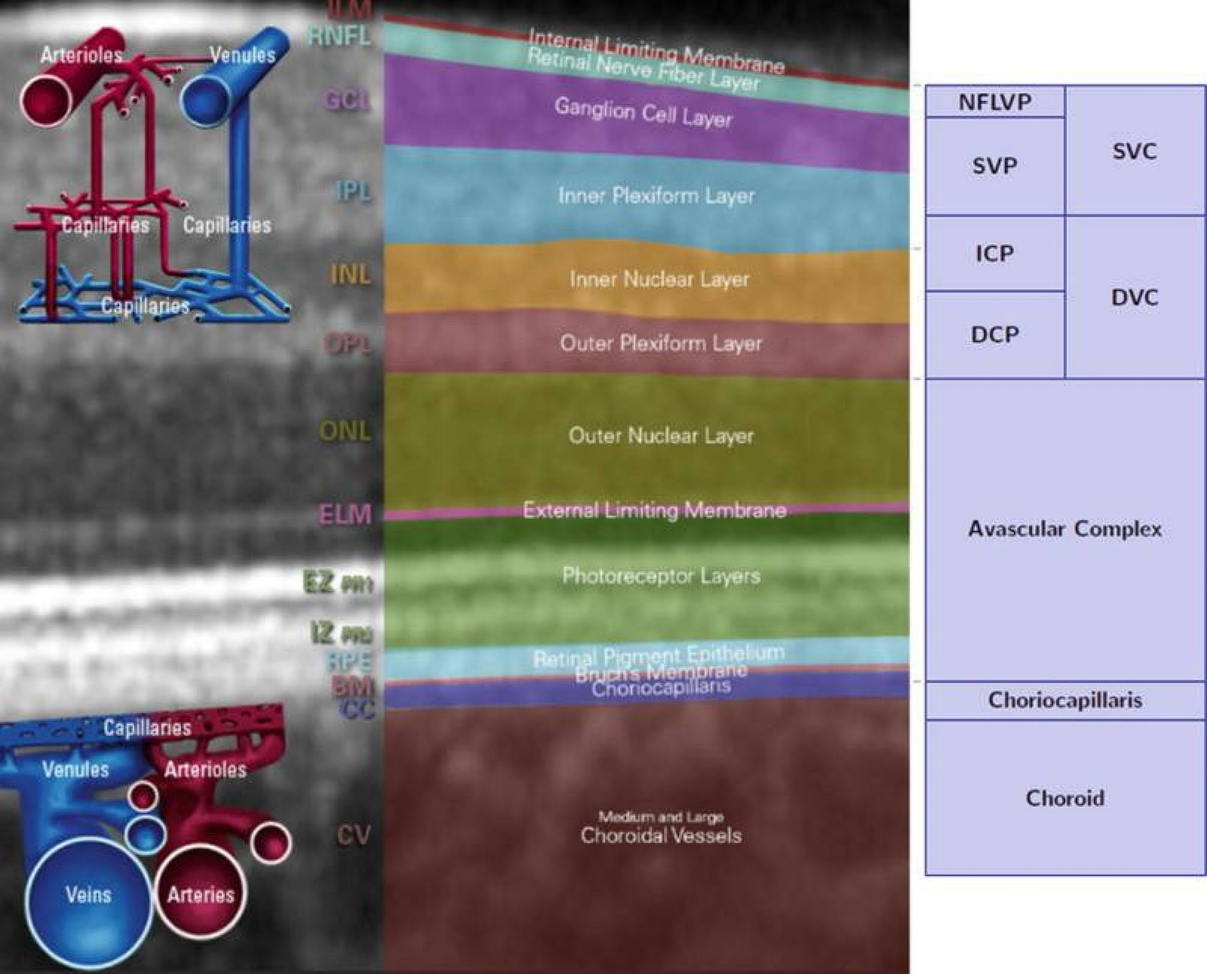
Left: Schematic figure of the layers and vessel networks in the human retina. Right: Schematic figure of the slab definitions. *SVC* superficial vascular complex, *NFLVP* nerve fiber layer vascular plexus (part of SVC), *SVP* superficial vascular plexus (part of SVC), *DVC* deep vascular complex, *AC* avascular complex, *ICP* intermediate capillary plexus (part of DVC), *DCP* deep capillary plexus (part of DVC), *CC* choriocapillaris. From: Chap. 6, OCT Angiography (OCTA) in Retinal Diagnostics Copyright

A 3 × 3 mm OCTA image centered on the fovea was obtained using Heidelberg Engineering’s 1060 nm swept-source (SS)-OCTA technology, featuring rapid scanning and automated segmentation of the superficial retinal layer. Additionally, Spectralis® OCT scans employed a 15º x 15º optic nerve head protocol, utilizing Spectralis® software for en face OCTA images of the superficial and deep vascular complexes. Segmentation defined the upper and lower limits of the superficial vascular complex (SVC) and deep vascular complex (DVC). SVC spanned from the internal limiting membrane to 17 μm above the inner plexiform layer, while DVC ranged from 17 μm above the inner plexiform layer to the outer plexiform layer’s extremity (Fig. 4).

### Statistical analysis

Statistical analysis was conducted using SPSS version 26.0 (IBM Corp, Armonk, New York, USA). Figures were produced using Excel (Microsoft®, Redmond, Washington, USA). Categorical variables were reported as percentages, while continuous variables were presented as mean ± standard deviation (SD). To evaluate the normality of the distribution for continuous data, the Shapiro-Wilk test was performed. Since the data was not normally distributed, the Friedman test was utilized to compare each variable across the different timepoints (before and after surgery). When statistically significant differences were identified by the Friedman test, post hoc multiple comparisons using Bonferroni correction were performed to determine specific significant differences. A *p* value of 0.05 or less was considered statistically significant.

## Results

In this study, 22 eyes from 22 keratoconus patients who underwent collagen cross-linking (CCL) surgery were analyzed. The mean age of the participants was 20.09 ± 6.11(range 12–34 years), including 59% male, and the mean intraocular pressure (IOP) before the surgery was 13.59 ± 2.85 mmHg (range 8–19 mmHg). Moreover, preoperative corneal topography (*Pentacam*®) showed a mean Kmax of 52.42 ± 1.15 D and a mean thinnest point of 480.36 ± 35.72 μm. In Table 1, patients’ demographic data and corneal topography analysis before surgery were reported.

**Table 1:** Demographic characteristics and corneal topography analysis before CCL surgery

variable	Value (*n* = 22)
Male gender, % Left eye, %	59% 63.6%
Age at surgery time (years), mean ± SD	20.09 ± 6.11
Pre operation IOP (mmHg), mean ± SD	13.59 ± 2.85
Pre operation corneal topography	
Sphere refraction(D), mean ± SD Cylinder refraction(D), mean ± SD Axis refraction(D), mean ± SD Kmax(D), mean ± SD Thinnest point(µm), mean ± SD	-2.35 ± 2.16 -3.92 ± 2 109.22 ± 85.13 52.42 ± 5.4 480.36 ± 35.72

n: number, D: Diopter, µm: micrometer, IOP: intraocular

-pressure, Kmax: maximal keratometry

**Fig. 5:**
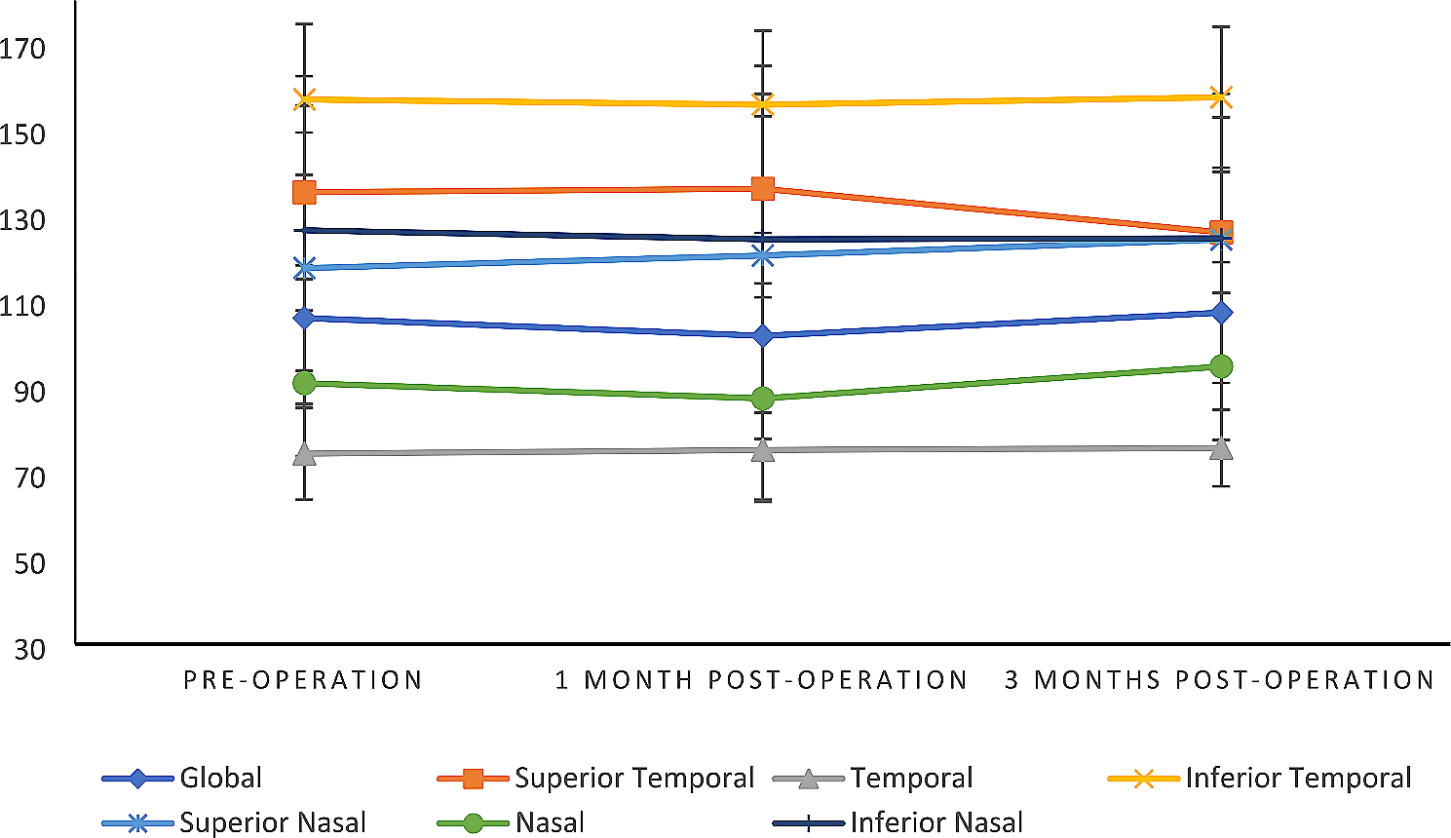
Peripapillary Retinal nerve fiber layer (ppRNFL) thickness changes in different regions prior to and following CCL surgery at 1 month and 3 months post-operation in patients with keratoconus (mean ± SD)

The mean of total, superior, and inferior retinal thickness was not significantly different before and after the surgery (*p* = 0.31, *p* = 0.13, and *p* = 0.25 respectively) (Table 2). Additionally, Peripapillary Retinal nerve fiber layer (ppRNFL) thickness in various regions revealed no marked variations across measurements conducted before surgery, one month after surgery, and three months after surgery (Table 2; Fig. 5).

**Table 2:** Comparison of average retinal thickness and peripapillary retinal nerve fiber layer(ppRNFL) thickness before and after the CCL surgery in patients with keratoconus (*n* = 22)

Variable	Pre-operation	1 Month post-operation	3 Months Post-operation	*P*-value*
ART(µm)				
total	78.63 ± 2.68	79.13 ± 2.69	80.13 ± 4.03	0.31
superior	79.18 ± 2.85	79.5 ± 2.87	80.9 ± 4.41	0.13
inferior	78 ± 2.76	78.68 ± 2.96	79.36 ± 3.84	0.25
Peripapillary RNFLT(µm)				
global	105.95 ± 12.32	101.81 ± 23.98	107.22 ± 11.77	0.28
superior temporal	135.27 ± 20.22	136.09 ± 22.07	125.9 ± 14.13	0.42
temporal	74.36 ± 10.73	75.22 ± 12.09	75.68 ± 8.94	0.5
inferior temporal	156.9 ± 17.55	155.68 ± 17.23	157.4 ± 16.39	0.77
superior nasal	117.59 ± 31.61	120.54 ± 32.46	124.27 ± 28.43	0.3
nasal	90.77 ± 16.94	87.22 ± 23.59	94.68 ± 17.16	0.08
inferior nasal	126.4 ± 35.93	124.31 ± 40.39	124.54 ± 33.69	0.48

ART: average retinal thickness, RNFLT: retinal nerve fiber layer thickness, (µm): micrometer

*Friedman test

*P*-values < 0.05 were considered as statically significant, all data were presented as mean ± standard deviation

Table 3. represents the amounts of macular OCT-A markers before surgery and at 1 month and 3 months post-surgery follow-up in all participants. In macular total vessel density (TVD) there were no significant changes in superficial vascular complex (SVC) and deep vascular complex (DVC) between timepoints (*p* = 0.075 and 0.17, respectively). However, the avascular complex (AC) revealed significant changes only between 1 month and 3 months after surgery (*p* = 0.031). For macular large vessel density (LVD), both SVC and DVC demonstrated significant differences between before surgery and the 3 months post-surgery follow-up (*p* < 0.001 and *p* = 0.002, respectively). Additionally, DVC showed significant changes between before surgery and 1month post-surgery, It decreased from 20.2 ± 4.3% to16.9 ± 5.1% (*p* = 0.048). Similarly, in macular capillary vessel density (CVD), SVC and DVC showed significant changes between before surgery and the 3-month follow-up (*p* = 0.031 and *p* = 0.02, respectively). In comparison to the macula, optic nerve head (ONH) OCT-A markers remained stable between timepoints for LVD and CVD. In TVD, the SVC and DVC showed no significant changes between timepoints (*p* = 0.55 and *p* = 0.66, respectively). The only significant change in the ONH was exhibited in the AC for TVD, which showed a difference between preop and 3 months post-surgery (*p* = 0.005) (Table 3).

**Table 3:** Comparison of macular and optic nerve head *optical coherence tomography angiography vessel density* in keratoconus patients before and after CCL surgery at 1 month and 3 months follow-up (*n* = 22)

	Pre-operation	1 Month post-operation	3 Months Post-operation	*P*-value*
Macula VD (%)				
TVD				
SVC	37 ± 3.3	36.4 ± 3	36.5 ± 3.9	0.075
DVC	44.4 ± 2.7	43.6 ± 2.7	44.8 ± 3.3	0.17
AC	50.6 ± 2.6	50 ± 2.1	49.1 ± 2.2	0.028§
LVD				
SVC	14.6 ± 6.6	12 ± 4.3	9.9 ± 5.4	0.001†
DVC	20.2 ± 4.3	16.9 ± 5.1	15.2 ± 6.7	0.002†&
AC	28.2 ± 2.4	27.1 ± 3.1	25.1 ± 4.2	0.11
CVD				
SVC	22.5 ± 4.1	24.4 ± 4.8	26 ± 6.2	0.028†
DVC	24.3 ± 4.5	26.8 ± 6.6	29.3 ± 8.6	0.022†
AC	23 ± 2.4	23.5 ± 3.4	24.8 ± 3.6	0.18
Optic nerve head VD (%)				
TVD				
SVC	45.9 ± 4.8	44.2 ± 4.4	45.5 ± 4.2	0.55
DVC	36.9 ± 3.2	36.4 ± 2.3	37 ± 1.5	0.66
AC	49.2 ± 2.2	48.9 ± 2.5	47.6 ± 1.7	0.005§
LVD				
SVC	33.3 ± 8.2	29.6 ± 9.4	32.7 ± 7.2	0.11
DVC	8.7 ± 3.4	8.9 ± 4.1	7.1 ± 3.1	0.1
AC	25.8 ± 6.1	27.5 ± 5.3	26.7 ± 4.6	0.094
CVD				
SVC	17 ± 9.7	16.2 ± 4.8	17.4 ± 6.3	0.28
DVC	28.2 ± 5.5	27.4 ± 4.9	28.9 ± 3.5	0.24
AC	25.7 ± 4.3	24.1 ± 3.3	23.3 ± 3.6	0.21

TVD: total vessel density, LVD: large vessel density, CVD: capillary vessel density, SVC: Superficial Vascular Complex, DVC: Deep Vascular Complex, AC: avascular complex

*Friedman test

*P*-values < 0.05 were considered as statically significant, all data were presented as mean ± standard deviation

Further statical analysis in *p*-value < 0.05 (statically significant) followed by post hoc with Bonferroni adjustment for multiple comparisons, §: statically significant comparison between 1month after surgery and 3 months after surgery, †: statically significant comparison between before surgery and 3 months after surgery, &: statically significant comparison between before surgery and 1 month after surgery.

## Discussion

Our study showed that although CCL does not affect retinal layer thickness, it may have effect on the perfusion of retinal layers at least in 3 months follow up. Corneal cross-linking (CCL) is generally considered a safe procedure, with the majority of reported complications typically confined to the cornea itself [8, 14]. Previous studies have shown that, when combined with riboflavin and a minimum corneal thickness of 400 micrometers, very little UV-A radiation reaches inner ocular tissues like the lens and retina during CCL [15]. This fact has raised concerns about the potential for retinal damage from UVA exposure during the CCL procedure. However, in vitro studies have demonstrated that riboflavin provides a protective shield, significantly reducing UVA transmission to inner ocular structures [12, 15, 16]. In this study, it was observed that the overall average thickness of the retina remained relatively stable before and after the CCL procedure, consistent with findings from Nasrollahi and Ozsaygili ‘s study [17, 18]. This stability can be attributed to the protective role of the cornea and crystalline lens, which absorb and shield the retina during CCL treatment [10]. However, it’s important to note that Barisan et al. reported different results from our study. In their study, which involved seventeen eyes of patients with keratoconus treated with CCL, they observed an increase in central macular thickness, indicating variations in the impact of CCL on retinal thickness compared to our findings [19]. Furthermore, in the study conducted by Mirzaei et al., they documented transient anatomical and functional changes following CCL, which contrasts with the outcomes of our study. This suggests that the effects of CXL on ocular structures can vary among different research investigations [9]. Moreover, it’s worth noting that the average retinal nerve fiber layer (RNFL) thickness across all areas exhibited no significant change pre- and post-operation, aligning with the findings in Ozsaygili et al.‘s study [18]. This similarity suggests that the integrity of the RNFL may be preserved during the CXL procedure. Regarding macular blood supply, notable changes emerged post-surgery, notably in the avascular complex (AC) of total vessel density (TVD) between one and three months. Significant alterations in large vessel density (LVD) and capillary vessel density (CVD) were observed in both superficial and deep areas by the three-month mark. Conversely, the optic nerve head (ONH) exhibited relative stability, aside from a marked shift in ONH total vessel density (TVD) within the AC between one- and three-months post-surgery. These distinctions emphasize distinct responses in macular versus ONH vasculature, highlighting region-specific adjustments following the surgical procedure. In our study, we observed vascular changes that contrasted with the findings of Barbisan et al. In our examination, diminishing vascular supply were evident, whereas Barisan et al. did not report any new vascular abnormalities in their fluorescein angiography results [19]. Our study has some limitations. First of all is the small sample size and the other is relatively short follow up period. Further investigations with Larger sample size and longer follow up period is suggested. Moreover, all of the participants of our study underwent standard Dresden protocol for CCL, so further studies, including various CCL methods, are needed to determine the definitive impact on blood supply.

## Conclusion

The use of UV rays in collagen cross-linking (CCL) appears to affect retinal thickness and blood supply. This study found reduced blood supply in the macula area post-CCL, potentially due to UV exposure. While retinal thickness fluctuated, long-term monitoring is crucial for assessing optic nerve and macula blood supply changes post-CCL, particularly in patients with conditions like diabetes.

## Data Availability

All data supporting this research are provided in the manuscript.
